# Translation and psychometric validation of the Persian version of palliative care attitudes scale in cancer patients

**DOI:** 10.1186/s12904-023-01223-3

**Published:** 2023-07-17

**Authors:** Sajjad Bagheri, Maasoumeh Barkhordari-Sharifabad

**Affiliations:** Department of Nursing, School of Medical Sciences, Yazd Branch, Islamic Azad University, Yazd, Iran

**Keywords:** Psychometric validation, Scale, Attitude, Palliative care, Cancer

## Abstract

**Introduction:**

To improve cancer patients’ quality of life, palliative care is necessary. The growth of palliative care, along with the assistance of the government and the collaboration of specialists, also relies on the knowledge and attitude of people. In Iran, there is no tool available to gauge patient attitudes about palliative treatment. The Persian version of the Palliative Care Attitude Scale (PCAS-9) was translated and psychometrically validated in this research among cancer patients.

**Methods:**

This methodological study was conducted in two stages: translation stage and psychometric validation stage. The method of translation was based on that proposed by Polit and Yang. Utilizing a qualitative approach, the scale’s face and content validity were investigated. 162 cancer patients who required palliative care based on expert diagnosis participated in the confirmatory factor analysis to establish construct validity. Stability and internal consistency provided evidence of reliability. The data was examined using SPSS18 and AMOS.

**Results:**

The “Palliative Care Attitudes Scale” translated well across cultures. Validity on both the face and the content was acceptable. Confirmatory factor analysis (CFA) revealed a good fit for the original three-factor structure. The intra-class correlation coefficient (ICC) was equal to 0.89, while the internal consistency (Cronbach’s alpha) reliability of the whole scale was equal to 0.77.

**Conclusions:**

Persian version of the “Palliative Care Attitudes Scale” was acceptable and adequate in cancer patients. Using this tool makes it easier to assess how patients feel about receiving palliative care and how well training sessions are working to change patients’ views.

## Introduction

The global concern of cancer is escalating quickly [1]. From the 112,000 cases reported in 2016 to the 160,000 cases anticipated in 2025, an increase of 42.6% is anticipated in the number of new cancer cases in Iran [2]. More people are living longer after receiving a cancer diagnosis due to more successful treatments, including those with progressive refractory cancer. The physical and mental health consequences of this could be detrimental [3, 4]. The patients’ quality of life and ability to perform activities of daily living (ADL) can be significantly impacted by symptoms such as anxiety, depressed mood, pain, fatigue, dyspnea, and anorexia [5–8].

Over the past 50 years, palliative care has grown as an interdisciplinary specialty to enhance the quality of life and care for cancer patients and their families [9]. Early palliative care initiation improves patient quality of life and survival rate for many patients with advanced cancer [10]. Families of patients who receive palliative care are more satisfied with the treatment [11]. The evidence also indicates that oncology teams’ care can be improved by the timely and prompt involvement of specialist palliative care teams [9]. Even so, only a small percentage of people with advanced diseases use palliative care [12–14]. Only 14% of those who require palliative care worldwide, according to World Health Organization (WHO) statistics, actually receive it [15].

The WHO lists misunderstandings about palliative care, cultural and societal hurdles, and a lack of public awareness of the advantages of this sort of treatment as some of the obstacles to inadequate access to this type of care [15]. Despite recent improvements in availability to this sort of therapy, identifying the reasons of underutilization of it at the patient level is useful [16]. The results of studies show that patients may avoid palliative care services due to lack of knowledge or negative attitudes towards palliative care, such as misperception of it as end-of-life care [17–19]. Iran is included in category “3a” in the 2020 WHO report. A country in this category is characterized by the development of palliative care activism that is still patchy in scope[20]. In addition to the backing of the government and the assistance of specialists, the growth of palliative care in a nation also rely on public knowledge and attitude [16]. Of course, an appropriate inventory with several attitude parameters is required in order to measure attitude [21].

To measure the attitudes of persons with cancer and other severe diseases toward palliative care, Perry et al. created the Palliative Care Attitudes Scale (PCAS-9). This scale consists of nine questions and three subscales that measure patients’ desire to seek referrals (behavioral subscale), their comprehension of the advantages of palliative care (cognitive subscale), and their fear of palliative care (emotional subscale). The scoring method of this scale is based on the Likert scale of five degrees. Higher scores reflect more favorable opinions regarding palliative care, which is how patients respond to the items. The advantages of The PCAS-9 is the easy scoring process, and self-report/interview-based measures. In addition, it consists of three subscales, which makes it easier to compare with others [16].

Despite the scale’s high reliability and validity, it is typically advised that each cultural context be taken into account when localizing the scale to ensure its validity. This study was conducted with the aim of translating, culturally adapting, and psychometrically validating PCAS-9 to be used both in research and practice because there was no such instrument found in Iran to examine patient attitudes towards palliative care, and on the other hand, the implementation of supportive interventions for the development of palliative care requires the existence of a valid and reliable measurement instrument to assess patient attitudes.

## Methods

### Study design

This methodological research studied the translation and cultural adaptation, and psychometric properties of PCAS-9.

### Study sample

Patients with cancer who were referred to Shah Vali Hospital and Shahid Sadoughi Hospital in Yazd, central Iran, made up the research population. With the aid of convenience sampling, participants were chosen. Reading and writing proficiency, study-participation openness, notification of the disease, and a specialist-diagnosed need for palliative care were the inclusion criteria for the study. Psychological issues, such as communication and cognitive disorders, and a lack of interest in participating in the study were the exclusion criteria.

In the stage of translation and cultural adaptation, two translators who were proficient in both Persian and English were used for forward translation, and two translators were used for back translation.

Using purposive sampling, 10 patients and 10 experts in the fields of palliative care and instrument development were chosen to determine the face validity and content validity, respectively [22].

In factor analysis, the general rule of sampling knowledge is that there should always be more subjects than variables. For each item on the instrument, 5 to 10 people are required to determine the construct validity [23]. Therefore, maximum of 90 participants were needed for the confirmatory factor analysis (CFA). The scale was distributed to 170 cancer patients. All of them participated in the study with a response rate of 100%. Ultimately, a total of 162 scale responses were eligible for analysis (a 95.3% eligible rate).

To check reliability, it is suggested to have between 15 and 30 people [24], and 30 cancer patients were chosen in this study using convenience sampling.

### Procedures

The researcher visited Shah Vali Hospital and Shahid Sadoughi Hospital in Yazd, central Iran, after securing the required permissions. The samples were selected using a convenience sampling method based on the inclusion and exclusion criteria. The research was carried out from July to November 2021. Intercultural translation and psychometric validation and were the two phases of this study.

#### Translation and cultural adaptation

After receiving permission from the creator of the PCAS-9, cross-cultural translation was conducted using the translation and cultural adaptation guide of Polit and Yang’s seven-step model [25]. Initially, the instrument was translated from English to Persian by two Iranian translators who were fluent in both the Persian and English languages and cultures. Afterwards, specialists analyzed the Persian translations in order to generate a single translation. In the subsequent phase, the Persian to English translation was done by two other translators who were fluent in both Persian and English, but who were unfamiliar with the instrument’s key phrases. Afterwards, the English translation was approved with the consultation of experts a via consensus group session. The final revised version was then sent to the instrument’s original developer for approval comments. She approved all the items; and no change was made to the items.

#### Psychometric validation

Next, face validity (qualitative), content validity (qualitative), construct validity (CFA), and reliability were assessed. In order to determine the instrument’s face validity, it was administered to ten patients and the items’ difficulty, ambiguity, and appropriateness were evaluated [26]. In order to establish the content validity, ten experts in the fields of palliative care and psychometrics of the instrument were asked to give their opinions about the relevance, necessity and representativeness of the items. After a comprehensive examination of the instrument, they provided comprehensive and written opinion [27]. Corrections were made by the current research group after a thorough analysis of expert opinions. Construct validity of the scale was examined using CFA with a sample size of 162 individuals.

Stability (test-retest) and internal consistency (Cronbach’s α coefficient) were used to measure reliability. Two-week intervals were used to administer the Persian version of the scale in order to assess its stability.

### Data collection instruments

In this study, two questionnaires were applied to collect data.

1. Demographic information questionnaire which involved age, gender, marital status, education level, and duration of illness.
2. The Persian version of PCAS-9 was includes 9 items and assesses patients’ perspectives on palliative care along three emotional, cognitive, and behavioral dimensions. There are three items in each dimension and scoring is done according to a 5-point Likert scale (from never to very much). The emotional dimension is scored inversely. Each dimension has a score of 3 to 15. The original version of this scale was created by Perry et al. (2020). They investigated and confirmed the reliability and validity of this instrument using three samples of people with cancer and one sample of people with serious non-cancer diseases [16].

### Data analysis

The data collected in our study were analyzed using the statistical software SPSS 18 (SPSS Inc., Chicago, IL, USA) and AMOS.

The Kaiser-Meyer-Olkin (KMO) test and Bartlett’s sphericity test were used to confirm the sampling adequacy. A KMO value higher than 0.5 is acceptable [22, 28], and Bartlett’s test of sphericity should be less than 0.05 [29]. X2/df, the goodness-of-fit index (GFI), the adjusted goodness-of-fit index (AGFI), the root mean square residuals (RMR), the normalized goodness-of-fit index (NFI), the incremental goodness-of-fit index (IFI), the comparative fit index (CFI), and the root mean square of error approximations (RMSEA) were used to evaluate the model’s fit [30]. Items with factor loadings of > 0.3 were considered acceptable [31].

Using Cronbach’s alpha and intra-class correlation coefficients (ICC), the reliability of a survey was determined. Cronbach’s alpha and ICC values more than 0.7 are acceptable for interpreting the findings [32].

## Results

A total of 170 questionnaires were distributed, and 8 responses were ineligible due to missing data. The eligibility rate was 95.3% (170/164). The average age of the participants was 51.62 ± 14.99 years, and the average length of their sickness was 42.69 ± 36.13 months, as shown in Table 1. The majority of participants were men (54.5%), married (88.4%), and bachelor’s degree holders (59.5%) (Table 1).

**Table 1:** Demographic characteristics of the participants

Variables		N (%)	Mean ± SD
Gender	Male	89 (54.9)	
	Female	73 (45.1)	
Marital status	Single	19 (11.7)	
	Married	142 (88.3)	
Educational Level	High school	96 (59.3)	
	Diploma	48 (29.6)	
	Bachelor & above	18 (11.1)	
Duration of the disease (in months)			42.69 ± 36.13
Age (in years)			51.62 ± 14.99

### Face validity

Examining the patients’ perceptions of each item revealed that all of the items were intelligible to the patients and none were unclear. Thus, no modifications were made in the items at this stage. Hence, the Persian form of this scale was deemed conceptually clear, acceptable, and adequate.

### Content validity

Five of the items (1, 3, 4, 5, and 9) were adjusted based on the comments of the experts about the relevance of the items to the desired notion, the use of acceptable diction and terminology, and the correct arrangement of phrases.

### Construct validity

KMO Measure of Sampling Adequacy and Bartlett’s of sphericity tests provide indication if the data are appropriate for CFA. KMO index was equal to 0.801. Bartlett’s test was significant (P < 0.001). These results indicated that the data set was suitable for factor analysis (Table 2).

**Table 2:** KMO and Bartlett’s Test

Kaiser-Meyer-Olkin		0.801
Bartlett’s Test of Sphericity	Approx. Chi-Square	846.425
	df	36
	Sig.	0.000

CFA was used to validate the concept and dimensions of the scale by AMOS. Table 3 displays the model’s fit indices, all of which supported the model.

**Table 3:** Results of the confirmatory factor analysis

Index	Model value	Acceptable fit
*χ* ^*2*^ */df*	1.98	< 3.00
GFI(Goodness of Fit Index)	0.94	> 0.90
AGFI(Adjusted Goodness of Fit Index)	0.89	> 0.90
RMR(Root Mean square Residual)	0.010	< 0.05
NFI (Normed Fit Index)	0.94	> 0.90
IFI(Incremental Fit Index)	0.97	> 0.90
CFI (Comparative Fit Index)	0.97	> 0.90
RMSEA (Root Mean Square Error of Approximation)	0.07	< 0.08

**Fig. 1:**
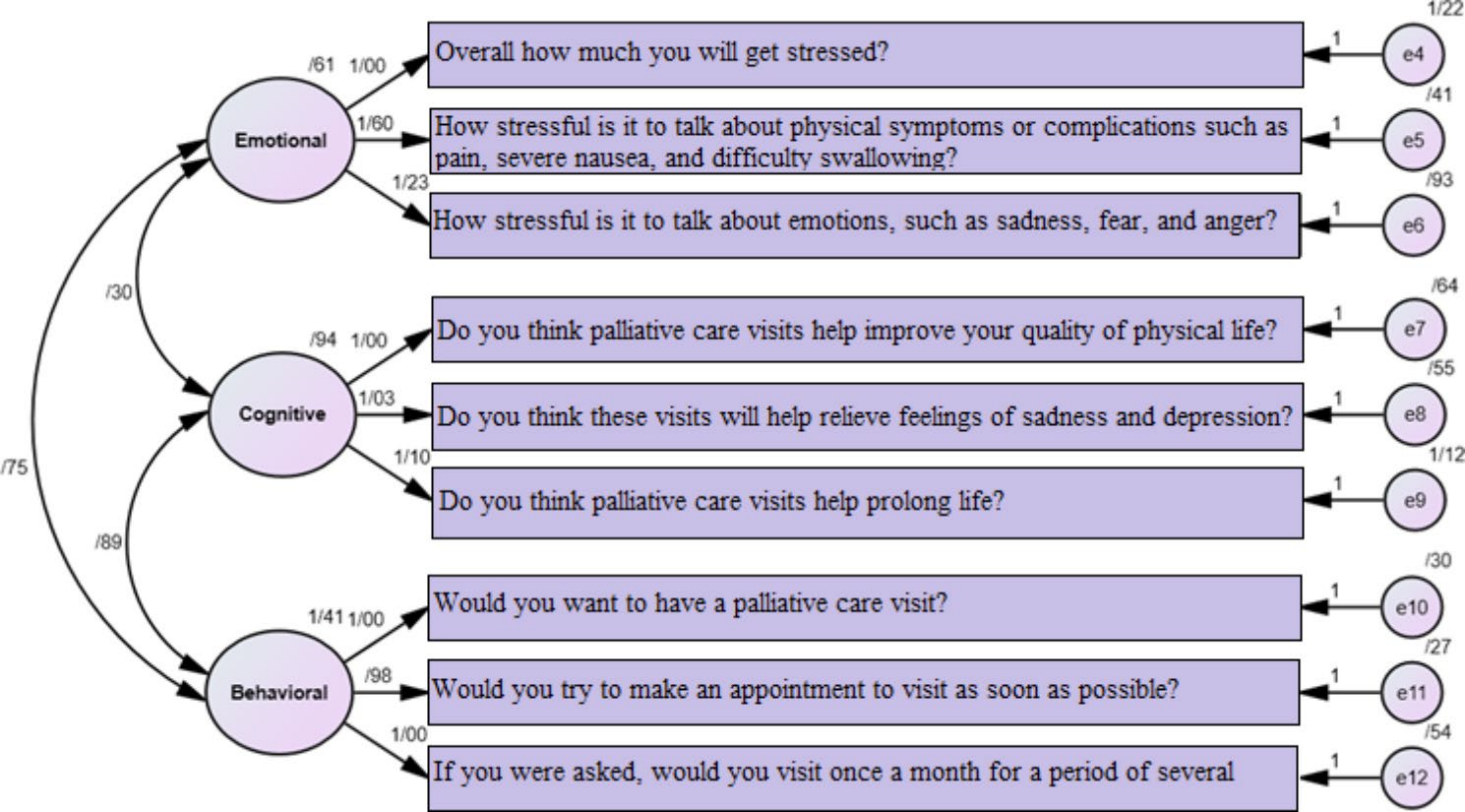
Confirmatory Factor Analysis for PCAS-9 (Persian version)

Figure 1 displays the model of dimensions of patients’ attitude towards palliative care in a standard and significant mode. Factor of emotional included items 1 to 3, factor of cognitive included items 4 to 6, and factor of behavioral included items 7 to 9. We assumed a correlation between all three factors. All indices and components have factor loadings greater than 0.3, and the membership of all researched factors in this variable has been verified (Fig. 1).

### Reliability

Internal consistency technique and ICC were employed to verify reliability. All subscales and the whole test had Cronbach’s alpha values more than 0.70, indicating that the subscales and the entire instrument had a satisfactory internal correlation (Table 4).

The test-retest reliability was calculated using the ICC coefficient for 30 patients with a two-week interval. The ICC for the emotional, cognitive, and behavioral dimensions of the patients’ attitude toward palliative care with a 95% confidence interval were 0.93, 0.94, and 0.83, respectively. As a result, it shows that the reliability of the questionnaire’s temporal stability or repeatability is acceptable and adequate (Table 4).

**Table 4:** Cronbach’s alpha and ICC for reliability of PCAS-9 (Persian version)

Factors	Number of items	Cronbach’s alpha	ICC
Emotional	3	0.76	0.93
Cognitive	3	0.82	0.94
Behavioral	3	0.92	0.83
Total	9	0.77	0.89

## Discussion

This study’s objective was to translate and psychometrically validate the Persian version of the “Palliative Care Attitudes Scale (PCAS-9)” among cancer patients. Using previously created instruments with strong psychometric qualities helps expedite and simplify cross-cultural research. Yet, these instruments must be culturally acceptable and have proper translation to be legitimate. For this reason, the translation process is an essential aspect of intercultural studies [33]. The results of this research revealed the acceptability of the translation of the original scale into Persian.

The findings of the face validity evaluation showed that the scale’s Persian translation was conceptually understandable, appropriate, and sufficient. The subject’s comprehension of the test idea and the difficulty, appropriateness, and ambiguity of the scale questions are the primary concerns in face validity [34]. According to several scholars, face validity is a component of content validity and the two are interdependent [35]. In this respect, the current study’s content validity was validated after several elements were changed in accordance with the advice of specialists.

Confirmatory factor analysis results validated the model’s fit. In other words, the Palliative Care Attitudes Scale measurement model has strong construct validity. The outcomes in the English version of the scale also demonstrated the model’s ability to fit various subgroups [16]. This scale’s emotional dimension is in line with the emotional dimension of the “palliative care perception scale” created by Milne et al., which measures how people feel about receiving palliative care [36]. The majority of studies stress the significance of attitude’s emotional components [37]. In the study by Milne et al., palliative care also includes a cognitive dimension [36]. This element refers to the opinions and viewpoints someone has regarding a particular topic [37]. The behavioral dimension of this scale includes actions or observable responses that are the result of an attitude toward a subject, which is consistent with the “behavioral factor of the palliative care competency framework” in the study by Connolly et al. [38].

Finding out if a scale is accurate and correct requires a reliability test [39]. The instrument’s stability and internal consistency were found to be adequate according to the reliability findings. The whole scale’s Cronbach’s alpha coefficient was 0.77. This coefficient in Perry et al.’s research was 0.84 [16].

One of the limitations of the current study was the use of a novel instrument created in English, which left the researcher struggling to find relevant materials for a better discussion. Since no test that evaluates the same or similar construct (palliative care attitude) was found in Persian language, convergent validity was not investigated in this study. Using convenience sampling is another limitation, therefore, caution should be taken in generalizing the results. In this research, due to the small number of items and the fact that scale content area was already specified, the content validity was only done using a qualitative method, and the content validity ratio (CVR) and content validity index (CVI) were not checked.

## Conclusion

The study’s findings showed that the Persian version of the “Palliative Care Attitudes Scale (PCAS-9)” is suitable and adequate for gauging Iranian cancer patients’ attitudes toward palliative care, according to psychometric results. Using this tool makes it easier to assess how patients feel about receiving palliative care and how well training sessions are working to change patients’ views. It is suggested to evaluate the validity of the scale in other clinical populations. The authors also suggest that future research explore the relationship between the PCAS-9 and clinical characteristics of the patients to explain the construct. Further research is needed to study the PCAS-9 scale in different languages and cultures.

## Data Availability

The datasets used and/or analyzed during the current study are available from the corresponding author on reasonable request.

## List of abbreviations

ADL: Activities of Daily Living
WHO: World Health Organization
PCAS: Palliative Care Attitude Scale
CFA: Confirmatory Factor Analysis
KMO: Kaiser-Meyer-Olkin
*χ*^*2*^: chi-squared
df: degree of freedom
GFI: Goodness of Fit Index
AGFI: Adjusted Goodness of Fit Index
RMR: Root Mean square Residual
NFI: Normed Fit Index
IFI: Incremental Fit Index
CFI: Comparative Fit Index
RMSEA: Root Mean Square Error of Approximation
SPSS: Statistical Package for the Social Sciences
AMOS: Analysis of Moment Structures
SD: Standard Deviation
ICC: Intra-class Correlation Coefficient
CVR: Content Validity Ratio﻿
CVI: Content Validity Index
